# Application of an Electronic Nose and HS-SPME/GC-MS to Determine Volatile Organic Compounds in Fresh Mexican Cheese

**DOI:** 10.3390/foods11131887

**Published:** 2022-06-25

**Authors:** Héctor Aarón Lee-Rangel, German David Mendoza-Martinez, Lorena Diaz de León-Martínez, Alejandro Enrique Relling, Anayeli Vazquez-Valladolid, Monika Palacios-Martínez, Pedro Abel Hernández-García, Alfonso Juventino Chay-Canul, Rogelio Flores-Ramirez, José Alejandro Roque-Jiménez

**Affiliations:** 1 Centro de Biociencias, Universidad Autónoma de San Luis Potosí, Carretera Federal 57 km 14.5, Ejido Palma de la Cruz, Soledad de Graciano Sánchez 78321, San Luis Potosí, Mexico; hector.lee@uaslp.mx; 2 Departamento de Producción Animal, Universidad Autónoma Metropolitana—Xochimilco, CDMX, Mexico City 04960, Mexico; mpalacios@correo.xoc.uam.mx; 3 Centro de Investigación Aplicada en Ambiente y Salud (CIAAS), Universidad Autónoma de San Luis Potosí, Lomas de San Luis 78210, San Luis Potosí, Mexico; loredldv@gmail.com (L.D.d.L.-M.); rogelio.flores@uaslp.mx (R.F.-R.); 4 Ohio Agricultural Research and Development Center (OARDC), Department of Animal Science, The Ohio State University, Wooster, OH 44691, USA; relling.1@osu.edu; 5 Facultad de Agronomía y Veterinaria, Universidad Autónoma de San Luis Potosí, Carretera Federal 57 km 14.5, Ejido Palma de la Cruz, Soledad de Graciano Sánchez 78321, San Luis Potosí, Mexico; anayeli.vazquez@uaslp.mx; 6 Centro Universitario UAEM Amecameca, Universidad Autónoma del Estado de México, Amecameca 56900, Estado de Mexico, Mexico; pedro_abel@yahoo.com; 7 División Académica de Ciencias Agropecuarias, Universidad Juárez Autónoma de Tabasco, Carretera Villahermosa-Teapa, km 25, R/A. La Huasteca 2^a^ Sección, Villahermosa 86280, Tabasco, Mexico; aljuch@hotmail.com

**Keywords:** e-nose, fresh cheese, VOCs, sensors, GC-MS

## Abstract

Electronic devices have been used to describe chemical compounds in the food industry. However, there are different models and manufacturers of these devices; thus, there has been little consistency in the type of compounds and methods used for identification. This work aimed to determine the applicability of electronic nose (e-nose) Cyroanose 320 to describe the differentiation of volatile organic compounds (VOCs) in fresh Mexican cheese (F-MC) formulated with milk from two different dairy cattle breeds. The VOCs were described using a device manufactured by Sensigent and Solid-Phase Micro-extraction (SPME) coupled to GC-MS as a complementary method. The multivariate principal components analysis (PCA) and the partial least squares discriminant analysis (PLS-DA) were used to describe the relationships of VOCs to electronic nose data, sensory data, and response levels. In addition, variable importance in projection (VIP) was performed to characterize the e-nose signals to the VOCs. The e-nose distinguishes F-MC prepared with milk from two dairy breeds. Sensor number 31 correlated with carboxylic acids most in F-MC from Jersey milk. The HS-SPME/GC-MS identified eighteen VOCs in F-MC made with Holstein milk, while only eleven VOCs were identified for F-MC made with Jersey milk. The more significant peaks in both chromatogram analyses were Propanoic acid, 2-methyl-, 1-(1,1-dimethylethyl)-2-methyl-1,3-propanediyl ester in cheese made from Holstein milk and Propanoic acid, 2-methyl-, 3-hydroxy-2,4,4-trimethylpentyl ester in Jersey milk cheese. Both compounds are considered essential carboxylic acids in the dairy industry. Thus, sensor 31 in the electronic nose Cyranose 320 increased its response by essential carboxylic acids identified by HS-SPME/GC-MS as a complementary method. The e-nose Cyranose 320 is potentially helpful for evaluating fresh Mexican cheese authentication independent of cows’ milk samples from different breeds.

## 1. Introduction

Cheesemaking is one of the most important industries in Mexico [1]. According to the Mexican Service Agrifood and Fisheries Information in 2021, the dairy industry uses approximately 35% of the total milk produced in the country [2]. Additionally, it is estimated that artisan cheesemakers use a similar percentage to make fresh Mexican cheeses (F-MC) [3]. Fresh cheeses are the most popular among all the Mexican cheeses, and almost 85% of this cheese is consumed in Mexico [3]. It is produced from raw milk employing a traditional manufacturing method and using a commercial rennet to give the characteristics of this type of cheese [1]. Most of the F-MC are made with milk from different dairy areas established in specific regions in Mexico, where the dairy breeds Holstein (H) and Jersey (J) stand out as the most common breeds on the farms [4]. Nevertheless, F-MC are little described, despite having a greater preference in the national market [1].

All cheeses have a unique aroma that may involve more than 600 volatile organic compounds (VOCs), such as short- or medium-chain fatty acids, alcohols, aldehydes, ketones, esters, or sulfur, which creates a characteristic smell and flavor of each cheese [5,6]. The VOCs have been reported as essential biomarkers to describe foods’ taste and shelf life [7]. Determining VOCs in dairy products may be critical to understanding their origins, evaluating differences in the aroma profile among similar cheeses, and ensuring dairy product safety and quality [8]. Research has linked milk VOCs to animal metabolism, interaction with the environment, diet, and management conditions [8,9]. Nevertheless, there is little information on how VOCs are related to dairy breeds, milk, cheesemaking, and potential benefits to consumer health [10,11,12]. Most of the information in the literature reports an abundance of VOCs in vastly varying concentrations since determining the volatile profile in complex food matrices is considered problematic due to different equipment and existing methods [7,12].

Recently, one way to analyze the aroma in foods is by using an electronic nose (e-nose) that can mimic the human olfaction system [5,13]. The e-nose is a gas sensor array that gives fingerprint response to specific volatile compounds, which can be used by pattern recognition algorithms [5]. Typically, the volatile molecules react with the sensing materials of the gas sensor and cause irreversible changes in electrical-related properties, such as conductivity [13]. These changes are then detected and characterized by pattern recognition algorithms to perform discrimination or classification [5]. In the food industry, e-nose has been used in dairy products to describe terms of flavor, variety type, ripening stage (in the case of cheese), or shelf-life prediction [3]. The e-nose systems have also been used to classify cheese type, production area, and ripening period [14]. Different electronic noses have classified Flamengo, Brie, Gruyère, and Mozzarella cheese [15]. According to Buratti et al. [16], e-nose analysis is a fast, easy, reliable, accurate, and non-polluting method. Nevertheless, different manufacturers have many electronic noses that use diverse numbers of sensors, and the results are inconsistent, with limited comparability between the different devices. Therefore, when an e-nose is used for the first time to characterize a type of food, the method must include an analyzing component using gas chromatography (GC) or gas chromatography-mass spectrometry (GC-MS) to classify the samples by their component patterns [5]. One technique for preparing samples for chromatographic analysis without using solvents is solid-phase microextraction (SPME). Since 1990 [17,18], the SPME method has been reported as an efficient technique applied in the food industry to estimate the concentration of aromas in different dairy products [19].

We hypothesize that e-nose Cyroanose 320 could have the capacity to evaluate the VOCs by its sensors responses to the aroma on F-MC elaborated with milk from two different dairy breeds. Thus, this study aimed to determine differences between sensor responses and to confirm the ability to evaluate differences of the VOCs in F-MC made with milk from different dairy cows’ breeds using an e-nose Cyroanose 320 and HS-SPME/GC-MS as a complementary method.

## 2. Materials and Methods

### 2.1. Cheese Samples

Two kinds of F-MC were chosen as cheese samples for e-nose measurement and HS-SPME/GC-MS VOCs identification. These cheeses are considered artisanal and protected by designation of origin according to the regulations that are required by the Mexican Federal Government (NOM-223-SCFI/SAGARPA-2018).

F-MC was sampled at two cheese factories in the central region of San Luis Potosi. These cheese factories use milk from the region’s two largest dairies, which employ purebred Jersey and purebred Holstein [20]. Briefly, on the “San Carlos Ranch”, 1800 Jersey cows are milked every day, and all the milk is collected in the same cold tank. Six hundred liters of milk from this tank are sold every day to the “Santa Maria” cheese factory (SMF) (21°47′56″ N; 100°44′14″ W). Holstein milk is from “Ojo de Agua Ranch” where 2000 Holstein cows are milked every day. Of the total milk obtained, 900 L per day are sold to the “Carrera-Torres” cheese factory (CTF) (21°46′18″ N; 101°44′51″ W).

For validation of the method, milk was evaluated to determine parameters of quality. We obtained twelve samples of milk that were collected directly from the milk cooling tank during the milking process. Immediately after sample collection, milk samples were analyzed following the manufacturer’s protocol for cow milk in a Lactoscan Ultrasonic Milk Analyzer (Milkotronic, Nova Zagora, Bulgaria) (Table 1).

In the cheese factories, the samples were obtained at the end of the cheese-making process, following the protocol described by Benedetti et al. [21]. Ten packets (500 g^−1^) from two lots of F-MC were randomly selected by the manufacturer at the beginning of their shelf life in each cheese factory. The cheeses were stored at 4 °C at a constant temperature until the next day’s analysis. All samples of F-MC were kept at room temperature (20 ± 1 °C) for 15 min before analysis. The F-MC manufacturing process and the ingredients used for its production were similar, as described by González-Córdova et al. [1].

### 2.2. Electronic Nose (E-Nose) Analysis

Analyses were performed using the Cyranose^®^ 320 (Sansigent, Pasadena, CA, USA), a portable electronic e-nose equipped with 32 functionalized-nanosensors incorporated into a matrix that adsorbs specific VOCs from the aroma of the two types of F-MC, causing an increase in the electrical resistance of each sensor [13]. Each sensor possesses different functions in the adsorption of VOCs, producing varying degrees of response due to their polymer composition, for example, polyvinyl butyral, polyvinyl acetate, polystyrene, and polyethylene oxide. The degree of response can be measured by the conduction of nanoparticles (black carbon and carbon nanotubes) [13,14].

**Figure 1:**
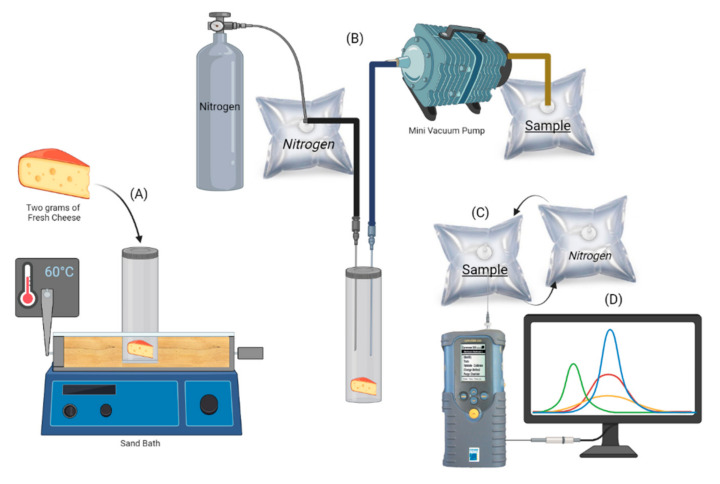
Diagram of the analysis two types of F-MC. (**A**) Incubation of F-MC sample for 15 min at 60 °C; (**B**) Absorption of VOCs in a Tedlar bag; (**C**) Data processing and pattern recognition. After each sample read, the e-nose required a purge using nitrogen gas (**D**).

For the aroma analysis of VOCs response by e-nose, the ten packets of SMF (Jersey milk) and CTF (Holstein milk) cheeses were sampled using six repetitions of each one. Two grams of each sample was placed in a 20 mL vial sealed with a silicone cap (Agilent^®^ 75.5 × 22.5, Santa Clara, CA, USA). Each vial was incubated at 60 °C for 15 min in a heating sand bath. After incubation, the needle of a vacuum pump (Millipore XX5411560, Burlington, MA, USA) punctured the silicone cap of the vials to supply the VOCs to three lines. The first line administered nitrogen gas, while the second line contained the VOCs in the F-MC sample. This gas mix was distributed to a Tedlar bag by the third line. Finally, the e-nose needle punctured the Tedlar bag to inhale the gas into the e-nose for reading. The analysis was performed as follows: 40 s of baseline purge (nitrogen gas), followed by 38 s of sampling; after 40 s, the lines were purged to prevent the sensors from acquiring VOC memory and to flush the lines. The device recorded the changes in electrical resistance of each of the 32 sensors due to VOC absorption into the sensors array (Figure 1). As a quality control, the baseline resistance of each sensor was recorded daily before and after the measurements (data not shown).

### 2.3. Solid-Phase Microextraction (SPME) Coupled to GC-MS

For identity analysis of the VOCs, the two types of F-MC were finely diced. Later, two grams of SMF and CTF cheeses were placed in a 20 mL vial sealed with a silicone cap (Agilent^®^ 75.5 × 22.5, USA). A 2 cm, 100 μm thick polydimethylsiloxane film (PDMS Stableflex SPME fiber (Supelco™, Sigma Aldrich, St. Louis, MO, USA) was used for sample preprocessing; the SPME fiber was activated at 270 °C following the manufacturer’s instructions. The PDMS fiber was exposed to the sample by continuous stirring at 60 °C for 15 min. The fiber was then analyzed by a gas chromatograph Agilent 6890^®^ coupled to a mass spectrometry detector Agilent 5975^®^ in electron impact ionization mode. The injection port was operated in splitless mode with a 0.75 mm glass wool liner. Injection port temperature was 220 °C; helium was used as carrier gas at a pressure of 36 psi with a constant flow of 0.9 mL min^−1^.

**Figure 2:**
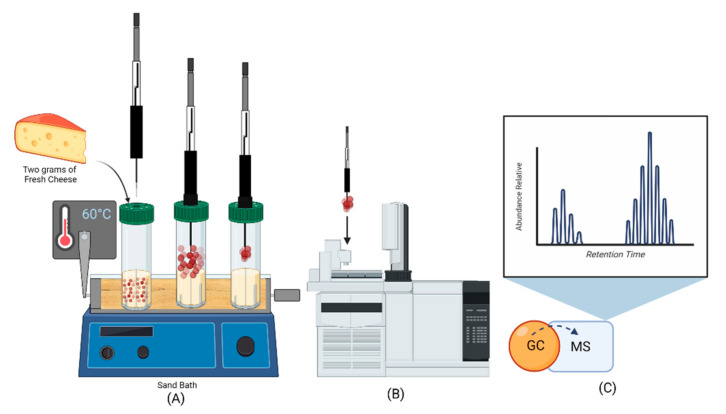
HS-SPME/GC-MS F-MC sampling procedure scheme. (**A**) External view for the steps of the extraction procedure, where the fiber is exposed to the VOCs. (**B**) SPME extraction and thermal desorption in GC injector. (**C**) Analysis and identification of the VOCs.

The chromatographic separation was performed through an HP 5MS (60 m × 0.25 mm × 0.25 μm) column (Agilent). The setting of the oven was as follows: 70 °C (initial; 0 min^−1^), 180 °C (10 min^−1^), 200 °C (5 min^−1^) for a run time of 15 min. The tune parameters were emission: 35 uA; energy: 69.9. The SCAN mode (50–500 *m/z*) was employed for identification of the compounds. The peak areas were taken to be the relative abundances of each volatile compound (Figure 2). The compounds were identified by comparison with standards in the NIST 14 library. Finally, the results were obtained and processed using Chemstation Software (Agilent, Santa Clara, CA, USA).

### 2.4. Statistical Analysis

The multivariate analyses principal components analysis (PCA) and partial least squares-discriminant analysis (PLS-DA) were performed using the increase in resistance of the 32 sensors obtained from the fractional difference: ΔR/Ro = (Rmax − Ro)/Ro; where Rmax is the maximum system response of each sensor, and Ro is the reference reading of each sensor (ultra-pure nitrogen).

The sum was normalized to reduce the environmental effect by dividing the response of each sensor by the sum of the absolute values of each sensor’s response, based on (∆R/Ro)i = (∆R/Ro)i/∑|∆R/Ro|j. Finally, a self-scaling was conducted to eliminate the effects of the sensor responses’ magnitude by subtracting the samples’ average from their response and dividing it by the standard deviation of samples. The analysis was performed employing the MetaboAnalyst 5.0 (Wishart Research Group, University of Alberta, CA, USA) online statistical software.

## 3. Results

### 3.1. Sensorgrams by E-Nose Response of F-MC Made with Milk from Jersey and Holstein Cows

**Figure 3:**
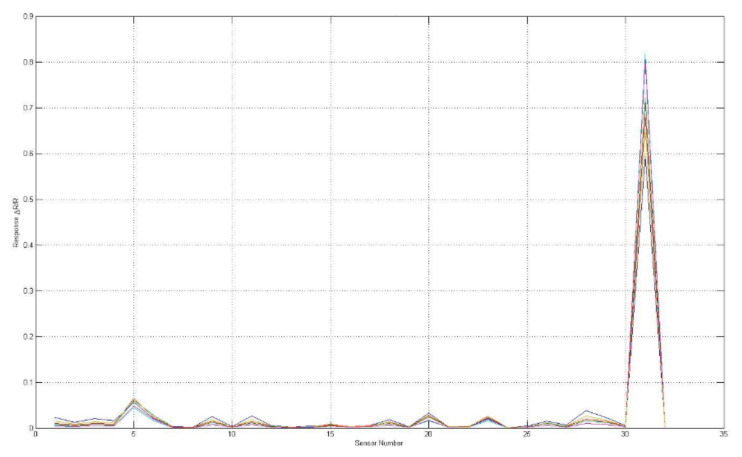
Sensorgram of the sensors’ responses to F-MC made with milk from Holstein cows (CTF).

**Figure 4:**
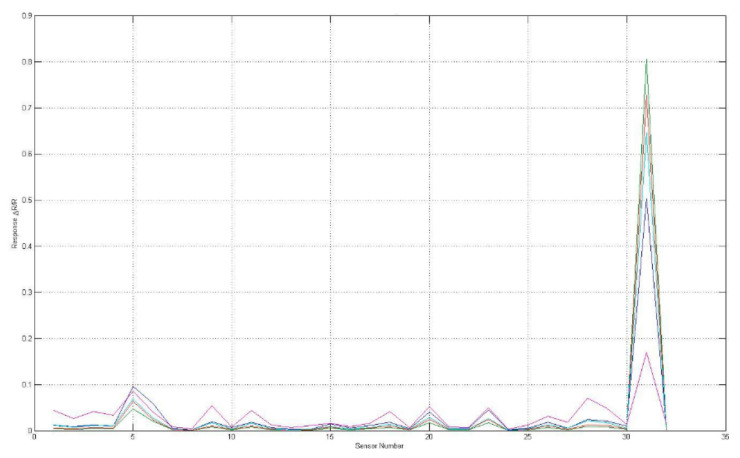
Sensorgram of the sensors’ responses to F-MC made with milk from Jersey cows (SMF).

The sensory response evaluated aromas of F-MC made with milk obtained from STF and CTF are shown in Figure 3 and Figure 4; the sensors (i.e., S5, S7, S8, S19, and S31) responded to the VOCs present in the sample of both kinds of F-MC. Interestingly, sensor number 31 showed greater sensitivity to the presence of VOCs than other sensors.

### 3.2. Principal Components Analysis (PCA) of F-MC Made with Milk from Jersey and Holstein Cows

**Figure 5:**
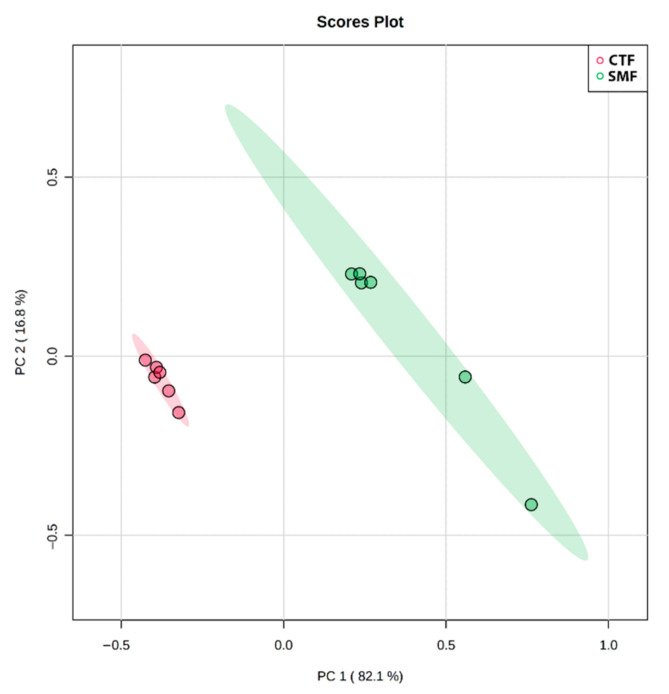
Principal component analysis (PCA) of VOCs with e-nose Cyranose 320 sensor response to two types of F-MC manufactured with milk from different dairy breeds. (Red indicates F-MC made with milk from Holstein cows, green represents F-MC made with milk from Jersey cows).

A PCA performed on selected variables in F-MC types from different dairy breeds provided a simplified overview of the relationship among their VOCs (Figure 5). Two principal components (PC1 and PC2) explained 98.9% of the total variance (82.1% and 16.8%, respectively). The score plot showed that F-MC from different breeds can be discriminated and that the latter two F-MC clusters are very similar in terms of their volatile compound content.

### 3.3. Partial Least Squares-Discriminant Analysis (PLS-DA) of F-MC Produced with Milk from Jersey and Holstein Cows

**Figure 6:**
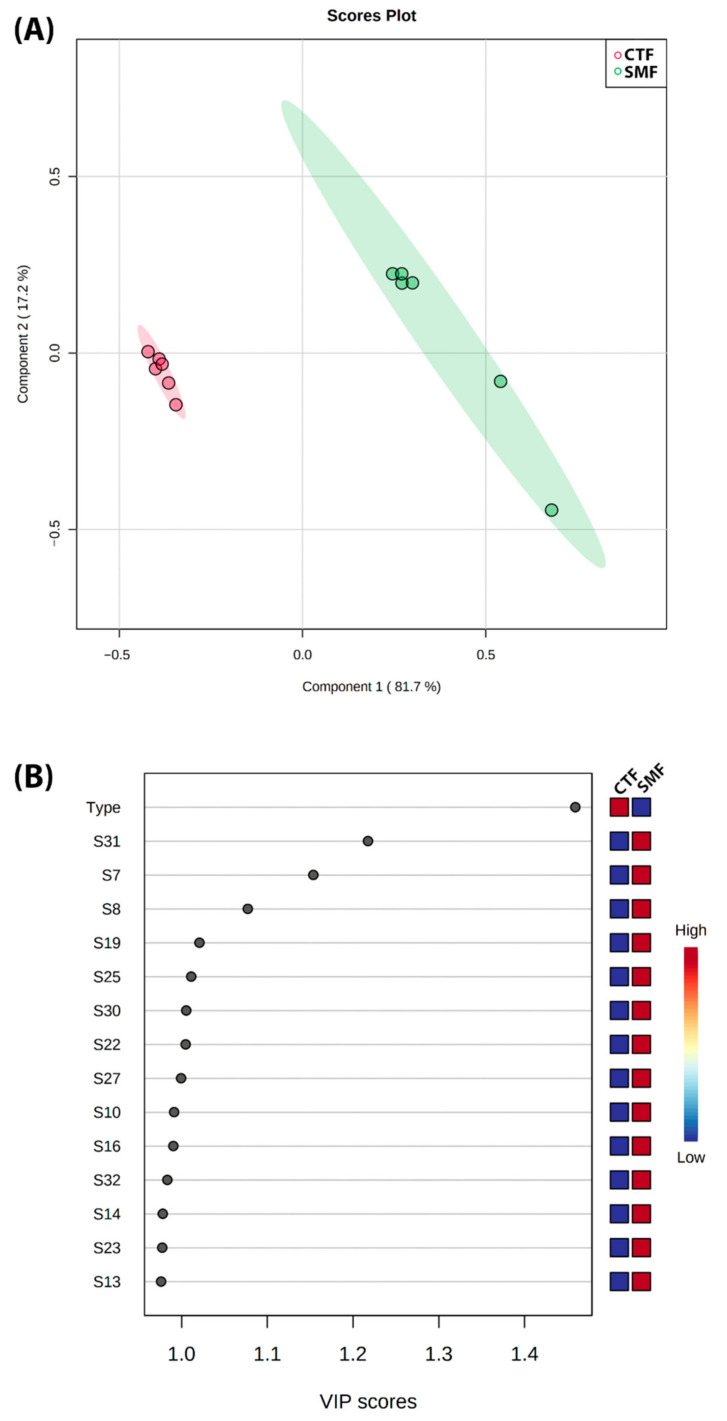
(**A**) Partial least squares discriminant analysis (PLS-DA) score plot in 2D graphs using the concentrations of all VOCs grouped by dairy breed. (**B**) Variable Importance in Projection (VIP) plots derived from the e-nose signals for the VOCs by two types of F-MC manufactured with milk from different dairy breeds (Holstein vs. Jersey).

The effect of variation on the VOC profiles and the concentration of specific metabolites in F-MC made with milk from different dairy breeds (Holstein vs. Jersey) revealed an ability to separate them and an affinity of the VOCs to the 32 sensors in the e-nose Cyranose 320. The VIP score shows the best correlation of sensors with the M-CF origin (milk). A higher VIP value is associated with SMF. The sensors that most correlated were 31, 7, and 8. In contrast, the CTF showed a low correlation with all the other sensors (Figure 6).

### 3.4. Hierarchical Clustering Analysis

**Figure 7:**
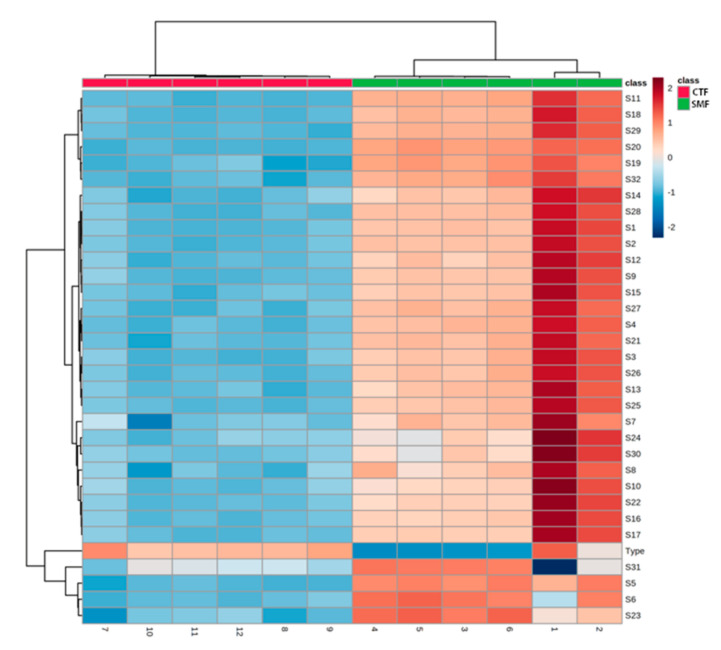
Heatmap of stable signals of e-nose sensors for six repetitions for each sample of F-MC produced with milk from two different dairy breeds (Holstein vs. Jersey). The blue frame indicates sensors with a negative correlation to the grade; the red frame indicates sensors with a positive correlation to the grade.

According to different correlation trends among the VOCs present in the F-MC made with milk from two dairy breeds (Holstein vs. Jersey) and the response signal intensity, all the sensors were subdivided into three categories: positively correlated, negatively correlated, and irrelevant. As shown in Figure 7, for sensors 10, 24, and 30, there was an apparent positive correlation between their signal intensity and the FC made using Jersey cow milk, while in the F-MC made with Holstein milk, there was an apparent negative correlation between sensor 24 and signal intensity.

### 3.5. Solid-Phase Microextraction Coupled to GC-MS

**Figure 8:**
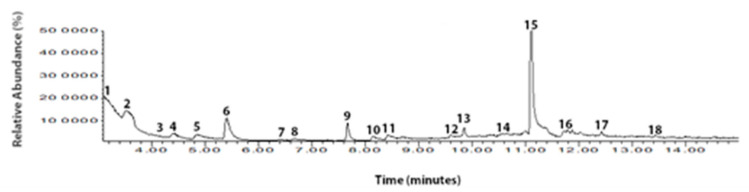
Total ion chromatogram of the VOCs and chemical composition in F-MC made with milk from Holstein cows. Chemical composition of CTF by HS-SPME/GC-MS with retention time^(rt)^: **1**. 1-Benzazirene-1-carboxylic acid, 2,2,5a-trimethyl-1a-[3-oxo-1-butenyl] perhydro-, methyl ester; **2**. Silicic acid, diethyl bis(trimethylsilyl) ester; **3**. 1H-Trindene, 2,3,4,5,6,7,8,9-octahydro-1,1,4,4,9,9-hexamethyl-; **4**. 4-Trimethylsilyl-9,9-dimethyl-9-silafluorene; **5**. Cyclotrisiloxane, hexamethyl-; **6**. Cyclopentasiloxane, decamethyl-; **7**. Methyl-[4-[2,6-dimethyl-3-[methylthio]-1,2,4-triazin-5(2H)-ylidene]-2-butenylidene]methylhydrazinecarbodithioate; **8**. Cyclohexasiloxane, dodecamethyl-; **9**. Propanoic acid, 2-methyl-, 2,2-dimethyl-1-(2-hydroxy-1-methylethyl)propyl ester; **10**. Hexane, 3-methyl-; **11**. Silicic acid, diethyl bis(trimethylsilyl) ester; **12**. Heptasiloxane, 1,1,3,3,5,5,7,7,9,9,11,11,13,13-tetradecamethyl-; **13**. Cycloheptasiloxane, tetradecamethyl-; **14**. Cyclodecasiloxane, eicosamethyl-; **15**. Propanoic acid, 2-methyl-, 1-(1,1-dimethylethyl)-2-methyl-1,3-propanediyl ester; **16**. Benzoic acid, 3,4-dichloro-, methyl ester; **17**. 1H-Indole, 2-methyl-3-phenyl-; **18**. 2,5-Cyclohexadien-1-one, 2,5-dimethyl-4-[(2,4,5-trimethylphenyl)imino]-.

**Figure 9:**
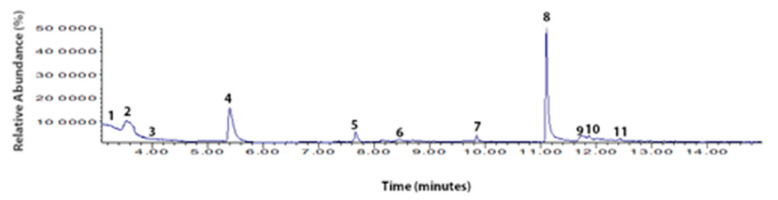
Total ion chromatogram of the VOCs and chemical composition in F-MC made with milk from Jersey cows. Chemical composition of SMF by HS-SPME/GC-MS with retention time(rt): **1**. Cyclotrisiloxane, hexamethyl-; **2**. Cyclotetrasiloxane, octamethyl-; **3**. 2-Methyl-7-phenylindole; **4**. Cyclopentasiloxane, decamethyl-; **5**. Cyclohexasiloxane, dodecamethyl-; **6**. 2-Hexen-4-ol, 5-methyl-; **7**. Cycloheptasiloxane, tetradecamethyl-; **8**. Propanoic acid, 2-methyl-, 3-hydroxy-2,4,4-trimethylpentyl ester; **9**. Nonahexacontanoic acid; **10**. 1,4-Dioxaspiro[4,5]decane-7-butanoic acid, 6-methyl-, 2-(methylsulfonyloxy)ethyl ester; **11**. 1-Monolinoleoylglycerol trimethylsilyl ether.

Volatile organic compounds are quite complex. Only a few compounds have a major effect on flavor development. Eighteen VOCs were detected in the F-MC made with Holstein milk (Figure 8). In contrast, in the F-MC produced with Jersey milk only eleven VOCs were identified (Figure 9).

## 4. Discussion

The current study evaluated the quality parameters of milk used to make the two types of F-MC. The milk quality results are consistent with different scientific reports [11,22], where the parameters of fat, protein, lactose, and total solids are similar to milk from the dairy breeds Holstein and Jersey [22]. Thus, the results of the applicability of e-nose Cyranose 320 in F-MC should be associated with its potential to evaluate the VOCs from cheese aroma by sensors’ response.

The e-nose showed sensitivity to the presence of the volatile chemical compounds emitted from F-MC manufactured with milk from Holstein or Jersey cows. Each line in Figure 4 and Figure 5 represents the response of individual sensors. The vertical axis represents sensor response, where: R_0_ is the resistance at baseline, and Rmax is the maximum resistance [6].

$$\frac{\Delta R}{R_{0}}=\frac{\mathrm{Rmax}-R_{0}}{R_{0}}$$

This response suggests that the e-nose can be used successfully to detect VOCs from fresh cheese [13]. Similar results have been reported by Abu-Khalaf et al. [14] and Fujioka [5], who used electronic devices to evaluate VOCs in dairy products. However, according to Ampuero and Bosset [23] and Mu et al. [24], using diverse electronic noses with various sensors and manufacturing protocols reduces the possibility of consistent comparison of sensor affinity to the types of VOCs. Additionally, Štefániková et al. [25] used an e-nose with two detectors to determine aroma profiles in Slovakian cheeses. They concluded that the aroma profile of cheese produced from cows’ milk has not been thoroughly investigated [25]. Thus, if a type of VOC is not identified, the results might not correlate with the results of other studies.

The first step of the e-nose analysis was to confirm the ability of the e-nose to distinguish between two types of F-MC made with milk from two dairy breeds (Holstein vs. Jersey). In this case, the analysis revealed the presence of VOCs in the F-MC samples. Additionally, two distinct groups were identified by PCA analysis (Figure 5). Related to our results, Falchero et al. [26] used an electronic nose to analyze milk from cows that grazed two different vegetation types in the Alpine region. They observed that their electronic nose grouped the sensors’ interactions depending on vegetation type. Finally, the authors concluded that the e-nose is helpful for rapid screening of profiles of milk volatile compounds, and they proposed analyzing the cheese produced with this milk for comparison with other reports. Our results show that the e-nose Cyranose 320 distinguishes the VOCs present in the two F-MCs [5]. According to our PCA analysis, the e-nose could identify the breed of dairy cow milk employed for cheese manufacturing, despite the sensors’ instability or affinity and the complex cheese aroma in some cases [26]; milk contains more than 100 chemical compounds such as water, fat, proteins, inorganic salts, and other primary compounds [12]. Yoo et al. [11] reported that in terms of sensory preference, milk from Jersey cows may be more appropriate for the use of an e-nose in the cheese industry [25]. Therefore, milk from the Jersey breed could contribute to dairy products’ diversification and provide better quality human nutrition [26].

Our research obtained e-nose sensor signals in response to the VOCs from two F-MC made with milk from Holstein or Jersey breeds. Sensors 31, 7, and 8 are more sensitive to F-MC made with Jersey milk (Figure 6). As we mentioned previously, there are different electronic devices (e-nose and e-tongue) for various applications and evaluations of aromas from food products [12,27]. Specifically, for the Cyranose e-nose, few reported studies in food science have described the affinity of its sensors [6]. Doty et al. [28] wrote an extensive description of the relative sensitivity of the response of the thirty-two individual sensors using purified chemical standards from each of five chemical classes (alcohols, aldehydes, amines, carboxylic acids, and ketones). Later, Doty et al. [28] correlated the chemical standards as a VOC source to each sensor’s response to describe which type of VOCs have a positive or negative correlation. Using the Doty et al. [28] description, our data obtained from F-MC made with Jersey milk (e.g., sensor number 31 that showed a greater response) could be linked to VOCs related to alcohols, ketones, or carboxylic acids present in the F-MC from Jersey milk. Now, the importance of our pilot study lies in that, although it is unknown which VOCs are responsible for increasing the resistance of some sensors, the response of sensor number 31 could be related to the cheese quality based on cows’ metabolism [11]. Ianni et al. [29] described that the catabolism-free amino acids represent the biochemical pathway that produces aldehydes, alcohols, carboxylic acids, amines, and sulfur compounds in cheese. Thus, a greater response by sensor number 31 may be related to better taste, color, flavor, and texture in the sensory analysis of fermented milk for cheese production [29].

Interestingly, in other scientific reports, the preference scores for different types of cheese such as Mozzarella cheese or Gouda cheese from Jersey milk have been lower in color, flavor, texture, taste, and general preference than cheeses made from Holstein milk [11]. Nevertheless, an important fact is that some sensor responses to VOCs representative of chemical classes in the cheese industry can be moderate or very low [6]. Hence, if the VOCs have little sensor response, these VOCs (such as carboxylic acids) may be undergoing changes in their activity, and the sensor could report a low signal intensity [13]. This low signal activity by the sensors may explain the apparent negative correlation of the F-MC made with Holstein milk (Figure 7) since SPME coupled to GC-MS demonstrated more VOCs in the F-MC manufactured with Holstein milk.

The HS-SPME/GC-MS method was applied to VOCs in the two types of F-MC made with milk from two different dairy breeds (Holstein vs. Jersey). As we mentioned before, greater amounts of fat and protein were observed in milk from Jersey cows than in Holstein milk. Thus, the VOCs identified for each F-MC are related to the nutrient contents in the milk source [11]. The analysis demonstrates that eighteen VOCs were detected in F-MC from Holstein milk, while eleven VOCs were reported in the F-MC made with Jersey milk (Figure 8 and Figure 9). Previous studies [19,30,31,32,33,34] provide similar evidence of the compounds found in both F-MC. However, according to Tunick et al. [19] and Contarini and Povolo [35], in dairy products, the SPME fiber detects different VOCs depending on incubation temperature, fiber exposure time, and the capacity of the cheese to melt [19]. Tunick et al. [19] concluded that a longer exposure time could cause the fiber to become saturated and lose volatiles (>30 min); so, a shorter time may be optimal for elucidating new compounds or VOC isomers [34]. In the current study, we observed that the highest peak in the two F-MC corresponded to different compounds. For F-MC from Holstein milk, propanoic acid, 2-methyl-, 1-(1,1-dimethylethyl)-2-methyl-1,3-propanediyl ester, was observed with the highest peak, and propanoic acid, 2-methyl-, 3-hydroxy-2,4,4-trimethylpentyl ester had the highest peak for F-MC made using Jersey milk. According to the National Center for Biotechnology Information, both compounds are carboxylic esters. Nevertheless, information is scarce, and both compounds have been reported just as derivates of dairy products [32,33,34]. The chemical food industry has reported that carboxylic acids are responsible for cheese fermentation and a slightly sweet flavor [32]. As a group, the role of the carboxylic acids in the cheese industry has different origins [32] since bacteria with fermentation capability in cheese [36,37] are related to cow metabolism, which is affected by the diet, which in turn affects nutrients for milk synthesis [35,37]. Thus, the present study suggests that the coupled HS-SPME/GC-MS method can elucidate e-nose sensor response to carboxylic acids present in different types of cheese and clarify which of them has a potential role, depending on the dairy breed and the quality of the milk. The results obtained can benefit Mexican government policies on labeling traditional products and increasing food sovereignty. Further research is needed to validate the results in commercial cheese samples.

Finally, this experiment allowed us to observe that e-nose Cyranose 320 has applicability in dairy product analyses through the response by its sensors to diverse VOCs. These sensors’ response is fundamental to discrimination between the chemical profiles of F-MCs. Additionally, applied to this discrimination of the chemical profiles, our study included the HS-SPME/GC-M, which allowed us to infer which compounds contribute to the discrimination between the groups, in contrast to the studies cited previously. We consider that the principal result of the current research is the development of accessible, fast, and selective detection of VOCs for the purpose differentiating between cheeses from different dairy breeds. This may have future application in the validation of artisan and commercial cheeses, cheese ripening, aroma perception, and cheese making recommendations.

## 5. Conclusions

This study revealed that the electronic nose with 32 sensors distinguishes global patterns and specific VOCs made with milk from the two most popular dairy breeds in Mexico (Holstein vs. Jersey). Only ten of the thirty-two sensors increased the response signal to the VOCs from the F-MC. Principal component analysis has shown a distinct grouping of the F-MC samples depending on the response of the sensors. Sensor number thirty-one can be related to specific VOCs in F-MC from Jersey milk. The main conclusion of this pilot study is that e-nose Cyranose 320 is a promising tool for fast and safe detection of VOCs involved in the aroma of F-MC. Nevertheless, the e-nose Cyranose 320 analysis still requires the HS-SPME/GC-MS method to elucidate which VOCs are in the cheese and establish a pattern depending on the specific sensor response to the VOCs. Thus, further research is needed on different types of Mexican cheeses from other milk breeds in different livestock systems to characterize the sensors’ response to their VOCs and establish a model with greater statistical robustness that allows it to be applied in the cheese industry.

## Figures and Tables

**Table 1:** Descriptive data on the composition of Jersey and Holstein cow’s milk.

Dairy Breed	Holstein	Jersey
Fat (%)	3.31	4.36
Protein (%)	3.42	3.91
Lactose (%)	4.40	4.79
Total Solids (%)	11.88	13.82

## Data Availability

The data presented in this study are available on request from the corresponding author. The data are not publicly available due to institutional instructions.
